# Mechanical test method and properties of a carbon nanomaterial with a high aspect ratio

**DOI:** 10.1186/s40580-016-0089-3

**Published:** 2016-11-07

**Authors:** Sang Koo Jeon, Hoon-Sik Jang, Oh Heon Kwon, Seung Hoon Nahm

**Affiliations:** 1 Department of Safety Engineering, Pukyong National University, Busan, 48513 South Korea; 2 Center for Energy Materials Metrology, Korea Research Institute of Standards and Science, Daejeon, 34113 South Korea; 3 World Tech Co., Ltd., Daejeon, 34368 South Korea

**Keywords:** Carbon nanotube (CNT), Carbon nanofiber (CNF), High aspect ratio, Mechanical properties

## Abstract

Superior nanomaterials have been developed and applied to many fields, and improved characteristic of nanomaterials have been studied. Measurement of the mechanical properties for nanomaterials is important to ensure the reliability and predict the service life times of products containing nanomaterials. However, it is challenging to measure the mechanical properties of nanomaterials due to their very small dimensions. Moreover, macro-scale measurement systems are not suitable for use with nanomaterials. Therefore, various methods have been developed and used to in an effort to measure the mechanical properties of nanomaterials. This paper presents a review of various evaluation systems and the measurement methods which are used to determine the mechanical properties of carbon nanotube (CNT) and carbon nanofiber (CNF), representatively. In addition, we measured the tensile strength and elastic modulus of the CNT and CNF in the scanning electron microscope (SEM) installed the nano-manipulator and the force sensor and this measurement system and results would be introduced in detail.

## Introduction

The nanotechnology has grown significantly over time and will continue to grown in the future. With the development of nanotechnology, a variety of devices and products containing nanomaterials have been manufactured and produced. Compared to bulk materials, nanomaterials generally have superior characteristic and performance capabilities, with the advantage of creating the smallest devices and products owing to the downsizing of materials. Representatively, carbon nanotube (CNT) and carbon nanofiber (CNF) are attractive for use in a variety of nanomaterials for which a high aspect ratio is required. Numerous attempts have been made to commerciallize these carbon nanomaterials in devices and products. In particular, CNT has been applied in numerous industrial areas, such as emitters [1], displays, x-ray and electron amplifiers [2–5], in the probe of scanning probe microscopy (SPM) [6], as a nano-balance and nano-tweezer [7, 8], memory devices [9, 10], lithium-ion battery [11–13], and even sensors for neurochemical detection [14–16]. Additionally, CNT was recently studied as a filler material to be mixed into a polymer/composite [17–19]. The areas in which CNF has been applied are similar to those of CNT. In relation to electrodes, CNF can be used in super capacitor [20, 21], lithium-ion battery [22–24], fuel cell [25, 26]. Several studies have shown that CNF can be applied as a gas absorbent [27, 28], catalyst support [29] and to compostie mixture [30–33]. However, most studies have focused on performance improvements for products with nanomaterials. The performance, durability and reliability of these product depends on the mechanical properties of nanomaterials. Therefore, it is necessary to measure the precise mechanical properties as a means of determining the reliability of products with nanomaterials, as the mechanical properties of theses materials are closely related to their reliability metrics, such as service life times of the products. But the mechanical behavior and the structure of bulk materials are different with nanomaterials due to the downsizing of materials and the manufacture processing. In other words, the mechanical properties of bulk materials could be not applied to nanomaterial production. Moreover the mechanical properties depend on the change of the physical and chemical characteristic in nanomaterials. Consequently, the mechanical properties should be directly measured on nanomaterials. The evaluation system and standard method of mechanical properties for bulk materials is established to determinate the performance and reliability of the general product with macro size. It is very important to establish the standard method and system, whereas those of nanomaterials have been not organized yet. But the many efforts have been attempted to researched to measure the mechanical properties of nanomaterials and present concretely in the next chapter.

The concepts which apply to the measurement of bulk materials are identical to those which apply to the measurement of nanomaterials, but the measurement of nanomaterials is very difficult, as nanomaterials cannot be controlled or be gripped with the hands and because loads and the strain levels at the nano level cannot be measured. For these reasons, a new system capable of measuring the mechanical properties of nanomaterials is needed. Consequently, for the precise measurement of the mechanical properties of nanomaterials with proper control a new and repeatable system is needed.

In this paper, we discuss various measurement methods and systems used with nanomaterials which have a high aspect ratio. Specifically, mechanical testing methods for CNT and CNF are reviewed and compared with each researches.

## Review

### Techniques for evaluating the mechanical properties of various nanomaterials with high aspect ratio

#### Bending test

**Fig. 1:**
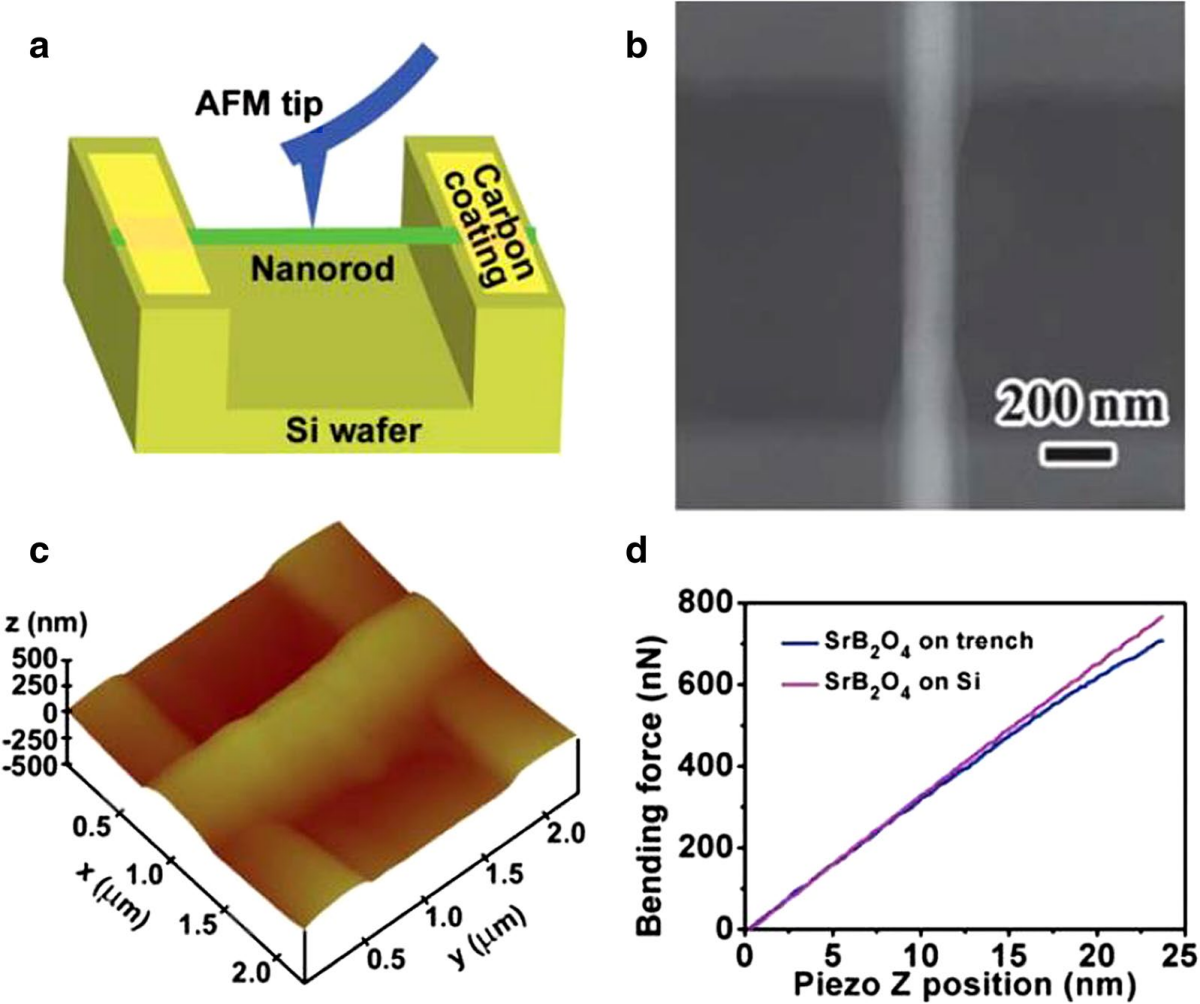
AFM three-point bending test on an individual SrB_2_O_4_ nanorod. **a** Schematic image of an EBID-fixed SrB_2_O_4_ nanorod in a three-point bending test with an AFM tip. **b** SEM and **c** AFM image of a fixed SrB_2_O_4_ nanorod suspended over the trench. **d** Representative bending force-piezo position (F-Z) curves of the SrB_2_O_4_ nanorod directly sitting on Si wafer and the SrB_2_O_4_ nanorod bridging a trench with both ends of the nanorod fixed [34]

Nanomaterials with high aspect ratio include nanowires, nanotubes, nanofibers, or nanorods. In this study, methods for measuring the mechanical properties of various nanomaterials with high aspect ratio, excluding carbon nanomaterials, such as CNT and CNF are described. Among the methods which can be used to measure the mechanical properties of these materials, such as the elastic modulus and breaking strength of nanomaterials with high aspect ratio, the most common is based on atomic force microscopy (AFM). AFM is also used to conduct bending tests on various nanomaterials with high aspect ratio, such as the three-point bending test [34–41] and the cantilever bending test [42–46]. As shown in Fig. 1, to carry out the three-point bending test, nanomaterials with high aspect ratio are placed across a trench-like substrate, with the AFM tip placed in the center of the nanomaterial. As the AFM tip is moved downward, the bending load applied to the nanomaterial can be measured. The trench-like substrate allows for sufficient bending deformation due to the bending load, as shown in Fig. 1a. To perform an accurate three-point bending test, as shown in Fig. 1b, the nanomaterials laid across the trench-like substrate should be fixed at both edges in order to prevent slipping when the bending load is applied. Owing to their size, nanomaterials cannot be fixed by traditional methods. Therefore, Pt, using a focused ion beam (FIB) and scanning electron microscopy (SEM), as well as carbon deposition can be used to fix the nanomaterial [46, 47]. To apply a bending load to nanomaterials using AFM, first the AFM contact mode is used to obtain an accurate image of the nanomaterial, as shown in Fig. 1c. This image is used precisely to position the AFM tip in direct contact with the center of the nanomaterial. When the AFM tip and nanomaterial are in contact, the bending load and bending displacement can be measured from the AFM tip. As shown in Fig. 1d, the bending load and bending displacement can then be calculated by the spring constant, deformation, and Z-axis shift displacement.

**Fig. 2:**
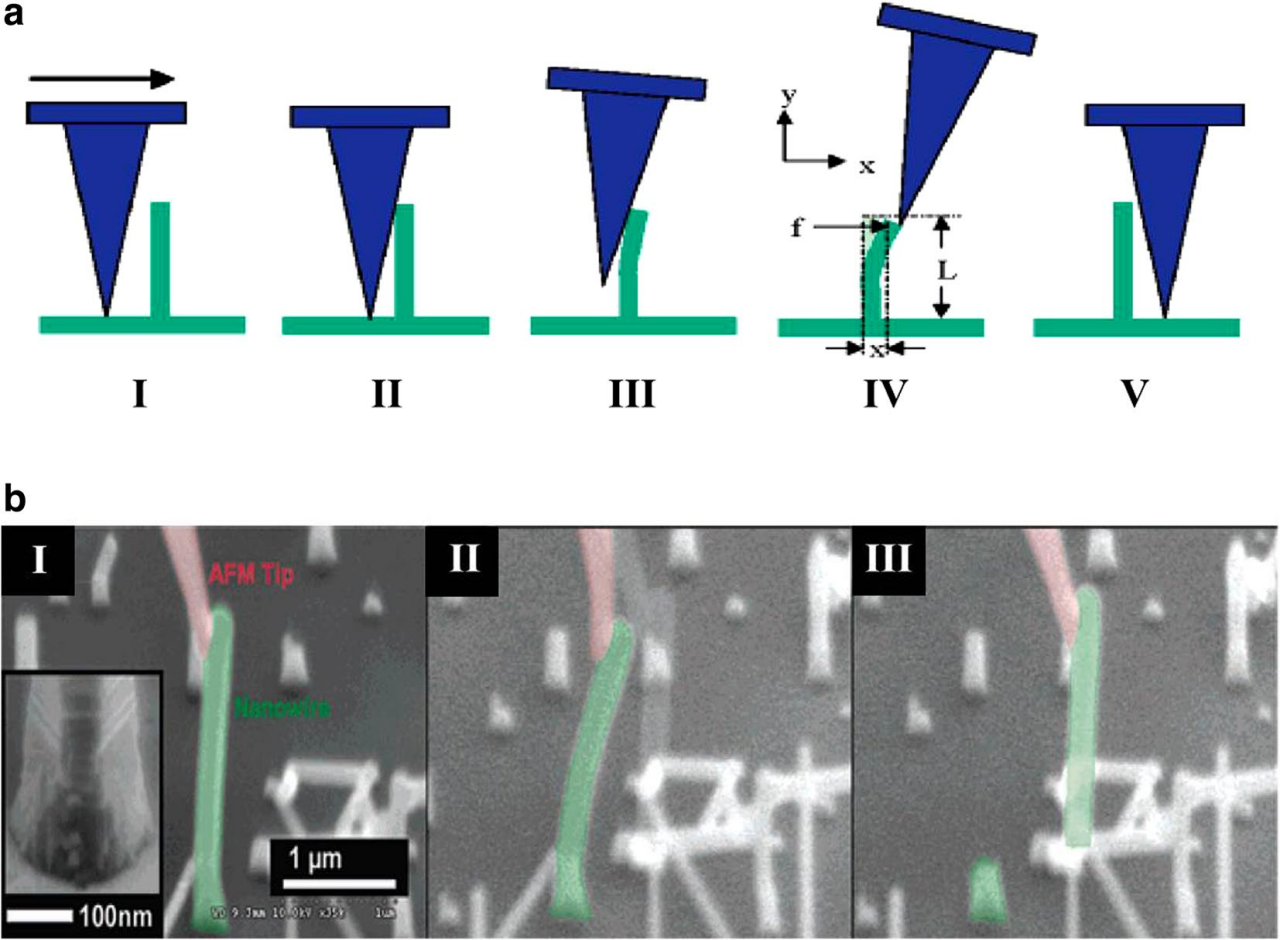
Cantilever bending test. **a** Procedure for measuring the elastic modulus of a NW in the AFM contact mode [42]. **b** Tilt corrected SEM images from a bending experiment. (**b**-*I*) NW with AFM tip before deflection. (**b**-*II*) Image just before fracture. AFM tip was bent, too. (**b**-*III*) Broken NW, the fracture occurred where the NW becomes thicker in good accordance with the FE simulations that indicate the maximum stress there. Mainly due to electron beam induced contamination deposition, the NW sticks to the AFM tip [43]

Another bending test method used to measure the mechanical properties of nanomaterials with high aspect ratio is the cantilever bending test. The cantilever bending test also uses the AFM tip, but two specific methods are utilized to measure the elastic modulus. Song et al. [42] as shown in Fig. 2a, measured the cantilever bending of an individual ZnO nanowire grown vertically on top of a substrate using the lateral force image and tomography information in the AFM contact mode (Fig. 2a I, II). The AFM tip is positioned close to the ZnO nanowire and the lateral force is measured while moving the AFM tip toward the ZnO nanowire. When the ZnO nanowire and AFM tip come into contact, the ZnO nanowire is bent, thus showing a linear increase in the lateral force (Fig. 2a III). In Fig. 2a IV, the deformation of the ZnO nanowire reaches a maximum, and the AFM tip crosses the ZnO nanowire. With further lateral movement of the AFM tip, the force on the ZnO nanowire returns to its initial value (Fig. 2a V). Hoffmann et al. [43] conducted another type of cantilever bending test on Si nanowires, as shown in Fig. 2b. This type of test is similar to the bending test described above in that it utilizes the AFM tip. However, in this case, the AFM tip is installed inside the SEM, and the bending of the nanomaterial is measured using the SEM image and not the signal from the AFM tip in order to calculate the elastic modulus. The AFM tip, which is attached to a nanomanipulator installed inside the SEM, is brought into contact with the Si nanowire (Fig. 2b I). With the AFM tip, the bending of the Si nanowire is triggered, eventually breaking the Si nanowire (Fig. 2b II, III). Immediately before the breaking of the Si nanowire, the deformation is measured by the SEM image. The elastic modulus, as well as the maximum bending stress, can thus be measured.

#### Nanoindentation

The test method that is most similar to the three-point bending test is the nanoindentation method, which is also based on AFM [48, 49]. Generally, the nanoindentation method is widely used for measuring the mechanical properties of thin film-like nanomaterials. By applying the AFM tip to the thin-film nanomaterials, their hardness and elastic modulus can be measured. The nanoindentation method can also be used to measure the elastic modulus of nanomaterials with high aspect ratio. The indentation method for nanomaterials with high aspect ratio is similar to the three-point bending test described above. The greatest difference between the two is that in the three-point bending test, the mechanical properties are measured by the tips of the nanomaterial at the edges of the trench, whereas in the nanoindentation method, the mechanical properties are evaluated with the nanomaterial laid on top of a flat substrate. In some studies, both the three-point bending test and the nanoindentation test were applied to identical nanomaterials so as to measure the elastic modulus, with the results of both tests then compared [39, 40].

#### Nano tensile test

**Fig. 3:**
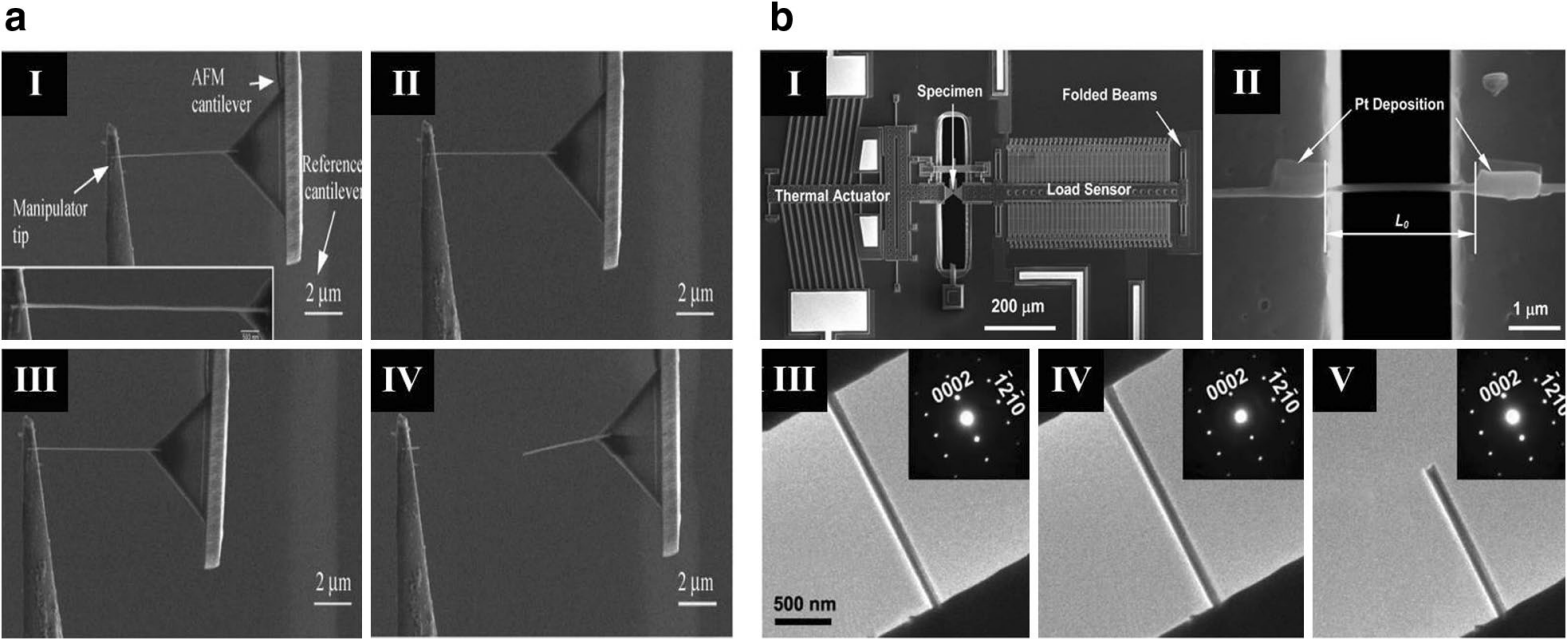
Nano tensile test. (**a**-*I–III*) A series of SEM images were taken during the tensile test for an NW with diameter of 20 nm. (**a**-*IV*) SEM image showing that fracture occurs on the NW when the load was applied to a certain value [50]. (**b**-*I*) SEM micrograph of microelectromechanical system used to test nanowires in situ a transmission electron microscope. (**b**-*II*) NW specimen suspended between thermal actuator and load sensor. Specimen ends were welded to the testing system by electron beam induced depositon of platinum. TEM images of a ZnO nanowire at various stages in the tensile testing: (**b**-*III*) before loading, (**b**-*IV*) at ~2.5% strain, (**b**-*V*) after failure [51]

The tensile testing of nanomaterials with high aspect ratio has been introduced in numerous papers [46, 47, 50, 51]. As shown in Fig. 3, either the AFM tip can be utilized or a nanoscale tension system can be established. The nanoscale tensile test is similar to the macroscale tensile test in that it applies a tensile load along the length of the nanomaterial. However, given that it is difficult to control the length and diameter of the nanomaterial, no clear regulations regarding the tension testing procedure have been prepared thus far. Figure 3a shows a method which can be used to conduct a tensile test which involves the installation of the nanomanipulator and AFM tip inside the SEM [50]. By fixing the ends of the nanomaterial on the respective edges of the nanomanipulator tip and AFM tip (Fig. 3a I), the AFM tip is slowly moved along the length of the nanomaterial, applying a tensile load (Fig. 3a II, III) eventually breaking the nanomaterial (Fig. 3a IV). The tensile load applied to the nanomaterial is calculated by the cantilever stiffness and deformation of the AFM, whereas the final elongation at fracture is measured from the SEM image right before the break. This method is very similar to the test method that will be explained in this study. Figure 3b shows a microelectromechanical system capable of analyzing the crystallizability of a nanomaterial inside a transmission electron microscope (TEM) in real time and testing its tensile strength at the same time (Fig. 3b I) [51]. First, the nanomaterial with a high aspect ratio is mixed with ethanol, dispersed on the TEM copper grid, and then dried. From the dried TEM grid, a filament of nanomaterial is selected, placed on top of the testing stage, and fixed at both ends by means of FIB and Pt deposition (Fig. 3b II). The microelectromechanical system is installed on the TEM grid and, by moving the test stage, a tensile load is applied to the nanomaterial, which eventually breaks (Fig. 3b III–V). The tensile load on the nanomaterial is measured by the load sensor and its elongation at fracture is measured by the TEM image.

#### Other tests

In addition to the abovementioned test methods, various other methods are used to evaluate the mechanical properties of nanomaterials with high aspect ratio. Because nanomaterials are very small, actual testing is difficult. Therefore, in many cases, the computational method is often used. Agrawal et al. [51] determined the changes in the elastic modulus as the diameter of nanowires increased by conducting a molecular dynamic simulation, whereas Fonseca et al. [52] measured the elastic modulus of amorphous nanowires using the Kirchhoff model. Sundararajan et al. [41] used ANSYS, a commercial analysis program, to evaluate the compression characteristics of nanobeams. In addition, buckling tests of nanomaterials have been conducted. Xu et al. [50] used an AFM tip and a nanomanipulator to measure the breaking strength and the elongation at fracture of individual ZnO nanowires, whereas Young et al. [53] conducted buckling tests in two different modes on ZnO nanowires with different diameters grown on Ga/glass substrates. In addition, Chen and Bai [54, 55] used electric-field resonant excitation inside the in TEM to measure the bending modulus of ZnO nanowire and nanobelt, whereas Huang [56] used same method to measure the mass of the nanoparticles of ZnO nanowire. The same method was used to evaluate the mechanical properties of nanomaterials with high aspect ratio. Finally, AFM was used to conduct three-point bending fatigue tests in order to measure the fatigue life time and fracture toughness of Si and SiO_2_ nanowires [41]. We confirmed that the various mechanical test method have been developed to measure the mechanical properties of nanomaterials.

### Mechanical properties and measurement method for carbon nanomaterials with high aspect ratio

Various ways to measure the mechanical properties of nanomaterials with high aspect ratio such as CNT and CNF, have been assessed, as described in the previous section. In this study, a direct tensile test was performed for individual CNT and CNF using a nanomanipulator and a force sensor to measure the mechanical properties. The method will be described herein in detail while also introducing various methods for measuring the mechanical properties of CNT and CNF and reviewing the results.

#### Mechanical properties and measurement method for carbon nanotube

Over many years, numerous methods have been developed to measure the mechanical properties of CNT. CNT can be largely divided into the multi-walled carbon nanotube (MWCNT) and single-walled carbon nanotube (SWCNT) types. The measuring methods can be divided into experimental methods and the analytical methods. Because the diameters of CNTs are on the scale of sub-nanometers, many methods have focused on measuring the mechanical properties using an analytical method. The three most common analytical methods are molecular dynamics (MD), molecular mechanics (MM), and density functional theory (DFT). Yao et al. [57] used MD to calculate the tensile strength of armchair SWCNTs, finding a value of 9.6 GPa, and the elastic modulus, at 3.62 TPa. Jin et al. [58] reported that the results of the MD simulation method matched the results of the experimental method applied to the measurement of the elastic modulus of SWCNTs. Bao et al. [59] used the second-generation reactive empirical bond-order (REBO) and Lennard–Jones (LJ) potentials, based on MD simulation, and calculated the elastic modulus of SWCNTs as 929.8 GPa. They also found that the elastic modulus of SWCNTs is influenced by the diameter and chirality of the nanotubes, whereas Liew et al. used the same method to evaluate the elastic moduli, Poisson’s ratio, yield strength, and maximum tensile strength of SWCNTs and MWCNTs.

**Fig. 4:**
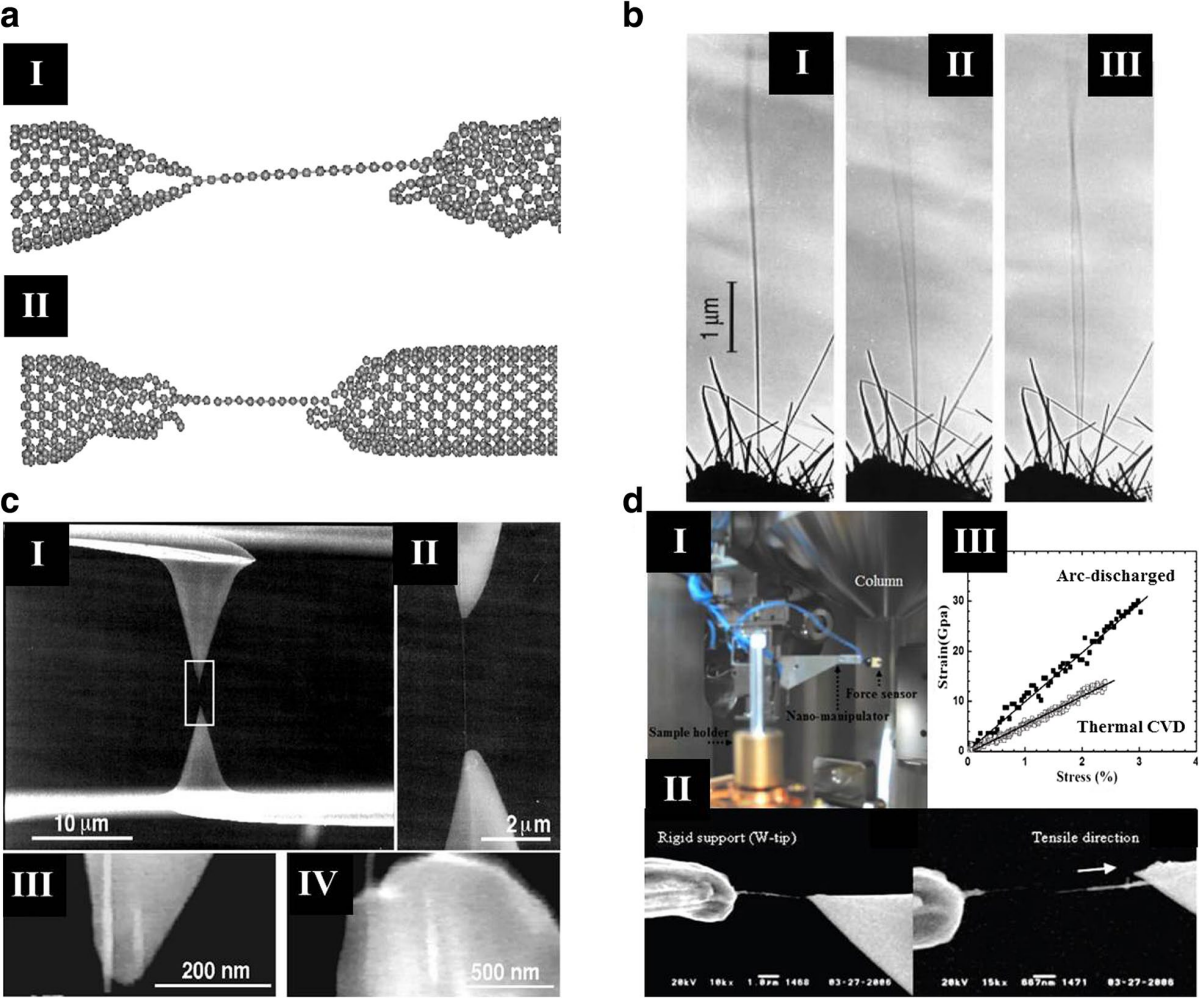
The various mecahical test of individual CNT. **a** Atomic chain of carbon nanotube when using Berendsen thermostat: (**a**-*I*) armchair structure, (**a**-*II*) zigzag structure [61]. **b** Nanotube response to resonant alternating applied potentials. (**b**-*I*) In the absence of a potential, the nanotube tip (L = 6.25 μm, D = 14.5 nm) vibrated slightly because of thermal effects. (**b**-*II*) Resonant excitation of the fundamental mode of vibration (V_1_ = 530 kHz). (**b**-*III*) Resonant excitation of the second harmonic (V_2_ = 3.01 MHz) [7]. **c** An individual MWCNT mounted between two opposing AFM tips. (**c**-*I*) SEM image of two AFM tips holding a MWCNT, which is attached at both ends on the AFM silicon tip surface by electron beam deposition of carbonaceous material. (**c**-*II*) High-magnification SEM image of the indicated region between the AFM tips. (**c**-*III*) Higher magnification SEM image showing the attachment of the MWCNT on the top AFM tip. (**c**-*IV*) Close-up SEM image showing the attachment of the MWCNT on the lower AFM tip [75]. **d** The CNT tensile test in the SEM. (**d**-*I*) Image of tensile test system inside SEM. (**d**-*II*) Individual CNT was appled to tensile load and fractured. (**d**-*III*) The results of CNT of the different growth method [79, 80]

They also found that MWCNTs break from the outermost layer in the initial stage, and that internal breaking arises later [60]. Moreover, they claimed that due to Stone–Wales defects, plastic deformation is generated, triggering brittleness. Mylvaganam et al. [61] used MD simulation and calculated the Poisson’s ratio and elastic modulus of armchair nanotubes as 3.96 and 0.15 TPa, respectively: and the Poisson’s ratio and elastic modulus of zigzag nanotubes were found to be 4.88 and 0.19 TPa, respectively, with a maximum elongation at fracture of 40%. Moreover, using MD simulation, the influence of defects, including vacancies, which can exist inside CNTs, on the elastic modulus and tensile strength of CNTs was investigated [62, 63]. Through molecular and solid mechanics simulations, the elastic moduli of SWCNTs and MWCNTs were calculated and found to be 1.1 and 1.6 TPa, respectively [64]. Meo et al. developed a finite element model based on MM simulation and calculated the tensile strength and the elongation at fracture of zigzag nanotubes as 94 GPa and 16.40%, respectively, and of armchair nanotubes as 123 GPa and 21.60%, respectively. They also found that the tensile strength and the elongation at fracture decreased if the CNTs possessed vacancy defects [65, 66]. To evaluate the mechanical properties of nanoscale materials with greater accuracy, a method based on the bonding force of atoms was developed. However, due to recent developments with regard of the use of simulations, general analytic tools were used to evaluate the mechanical properties of the nanomaterial. Ghavamian et al. used the finite element method to evaluate the mechanical properties of SWCNTs and MWCNTs. The influence of defects due to the vacancy or substitution of Si atoms on zigzag and armchair nanotubes was evaluated, and the mechanical properties, including the shear factor and elastic modulus, of the MWCNTs and SWCNTs were evaluated. The authors claimed that the finite element method is an appropriate method for evaluating the mechanical properties of CNTs [67, 68]. In addition to the abovementioned methods, the tight-binding method [69] and a self-consistent charge-density functional tight-binding (SCC-DFTB) method [70] have been used to measure the mechanical properties of CNTs, and MD, MM, and DFT together were used to compare the mechanical properties of CNTs and the changes in the mechanical properties caused by defective factors [71, 72]. To measure the mechanical properties of CNTs experimentally, the resonance method has also been used. Krishnan et al. [73] used TEM to measure the thermal vibration and calculated the bending elastic moduli of SWCNTs and MWCNTs as 1.25 and 1.8 TPa, respectively. Similarly, Wang et al. as shown in Fig. 4b, created resonance by applying an electric voltage to a CNT, where one end was fixed and the other end free. When the externally applied voltage frequency matched the natural frequency of the CNT, the resonance and frequency could be measured. The measured resonance was dependent on the bending modulus, length, area, and on other factors related to the CNT: thus, bending modulus of the CNT of 1.2 TPa was derived [7, 74]. Measuring the mechanical properties of very small CNTs has often been attempted using an AFM tip, as described above. Yu et al. [75] attached both ends of a single MWCNT filament to two AFM tips, as shown in Fig. 3c. They then moved the AFM tips apart to obtain an accurate tensile test of a single-filament MWCNT. The test resulted in an elastic modulus ranging from 0.25 to 0.95 TPa. Salvetat et al. used AFM and a specially manufactured substrate to measure the elastic modulus and shear factor of an SWCNT rope. They reported that the elastic modulus decreased as the crystallizability of the exterior wall of the MWCNT decreased [76, 77]. Demczyk et al. [78] manufactured a test stage using AFM to perform tensile and bending tests, resulting in an elastic modulus of 0.9 TPa.

We reported that a tensile test was conducted to measure the elastic modulus and tensile stress of MWCNTs [79, 80]. To observe the nanomaterial, SEM (TOPCON-300) was used, and a jig for attaching the nanomaterial inside the SEM, as well as a nanomanipulator (Klocke) for controlling the nanomaterial, were installed at the top inside the chamber, as shown in Fig. 4d I. The nanomanipulator was driven by a piezomotor and could move around all three axes to a maximum distance of 120 mm. To measure the load applied to the nanomaterial, a force sensor (Klocke) similar to an AFM tip was attached to the front side of the nanomanipulator. By using the attached force sensor, a single MWCNT filament selected from among a bundle of MWCNTs was attached to the force sensor using carbon deposition and was drawn out from the bundle. The carbon deposition process was continued until the surface of the MWCNT bundle was sufficiently covered. For firm fixation, carbon deposition was continued for 1 h. The end of the MWCNT filament that was drawn out was attached to the jig such that both ends of the MWCNT were firmly fixed. As shown in Fig. 4d II, the force sensor was moved along the length of the MWCNT such that the MWCNT received a tensile load and eventually broke. Figure 4d III shows the stress–strain curve of an MWCNT grown by the arc-discharge method and an MWCNT grown by the thermal CVD method, indicating that the mechanical properties of MWCNTs vary depending on the growth method. Moreover, the rate of resistance change regarding the tensile deformation of the CNT was measured in this study [81]. In this case, instead of a force sensor, an Au-coated tungsten tip was attached to the nanomanipulator. Feedthrough was used to connect the tungsten tip installed inside the SEM to an external measurement program and multimeter. By utilizing the same method, the MWCNT was fixed. While moving the tungsten tip along the length of the MWCNT, tensile stress was applied, increasing the electric resistance. The electric sensitivity of the MWCNT was thereby calculated.

#### Mechanical properties and measurement method for carbon nanofiber

**Fig. 5:**
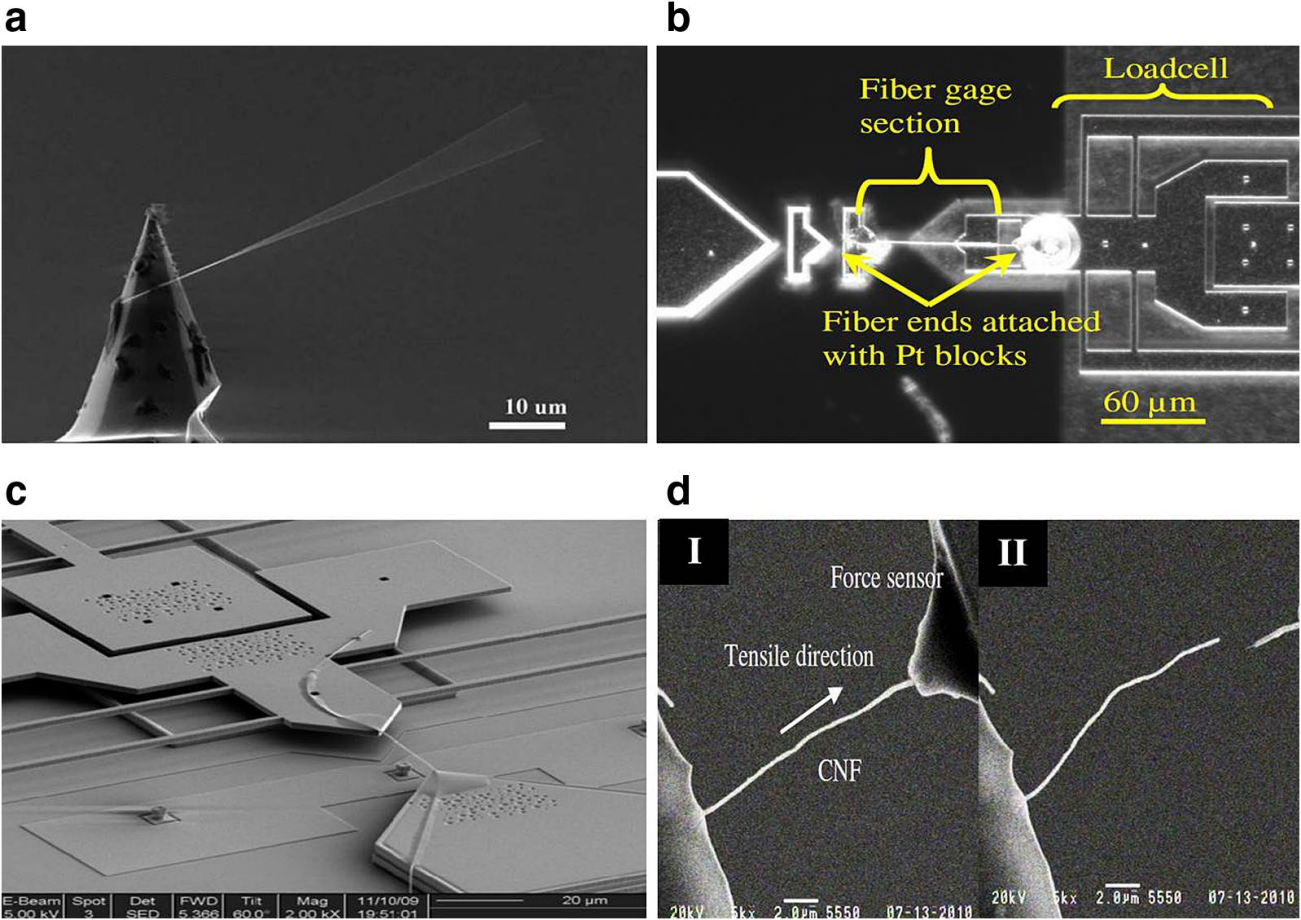
The various mechanical test of individual CNF. **a** SEM image of a mechanically resonating nanofiber clamped to an AFM cantilever tip [84]. **b** VGCNF tested by the MEMS-based mechanical property measurement platform [85]. **c** PAN nanofiber mounted on a MEMS loading platform [86]. **d** The SEM image of (**d**-*I*) before tensile test and (**d**-*II*) after tensile test [88]

CNF are most commonly manufactured by chemical vapor deposition (CVD) and electrospinning method, electrospun, and CNF finally can be grown through a stabilization and carbonization process. The various and complex structures of CNF are formed according to the manufacturing method. The mechanical properties of CNF were measured by the previous described method for nanowires and CNT. Compared to CNT, the mechanical properties of CNF were mostly measured using experimental methods. Wei et al. [82] reported that the mechanical properties can be calculated for three types of CNF which have single or multishell nanocone or cone-stacked structure using molecular dynamics simulation and revealed the properties depended on the structure. Figure 5 shows the experimental method used to measure the mechanical properties of CNF. Zussman et al. and Jacobsen et al. measured the elastic modulus of CNF using the resonance method, as shown in Fig. 5a, and the elastic modulus of CNF was respectively 680 and 68 GPa [83, 84]. These results were attributed to the different manufacture processes and structures. Therefore, the elastic modulus of CNF could be not defined as a unique characteristic value. Figure 5b, c show the tensile test system of CNF. A MEMS-based mechanical testing platform was design to perform the tensile test of CNF by Ozkan et al. [85]. As shown in Fig. 5b, vapor-grown CNF was put on the test device and the both ends of CNF were fixed by means of Pt deposition. The force and strain of CNF were measured by a load cell and CCD camera, respectively. Similarly, Arshad [86] created a nano-tensile tester using MEMS process, as shown in Fig. 5c and conducted a tensile test of the electrospun CNF via SEM. They reported changes of the tensile properties according to the temperature and the time of the heat treatment. Beese et al. [87] performed a tensile test of CNF using a nano-tensile stage which served to measure the mechanical properties of CNT in TEM, where the fracture mechanism was observed in detail. We also performed the tensile test of CNF using the method described in the previous section, as shown in Fig. 5d [88, 89]. After the CNF was dispersed on a cut TEM grid, the CNF was selected on the cutting plane. The CNF was vertically aligned to the cutting plane of the TEM grid using nanomanipulator, and the contact area of the CNF and the TEM grid was fixed by carbon deposition. The opposite of CNF was fixed on the force sensor and the tensile test was then performed. We also calculated the electrical sensitivity of CNF through the electrical resistance changes during the tensile test [89].

## Conclusions

In this paper, mechanical tests of CNT and CNF, a carbon nanomaterial with a high aspect ratio, were reviewed and various mechanical test methods were introduced. Due to the size limit of nanomaterials, the mechanical properties are evaluated by AFM and simulation methods. Recently, a nano-stage enabling the tensile testing of nanomaterials was designed through MEMS process development. The mechanical properties of CNT and CNF were evaluated in many studies but the research on CNF has been insufficient as compared to that of CNT. It was predicted that the mechanical properties of CNF were dependent on the various structures and manufacture processes used. Instead, CNF was mostly used as a composite material with the filler, with numerous mechanical properties for the composite materials reported. It is possible to evaluate the reliability and safety of nano-products using the developed method, and there is a need to measure the mechanical properties of more nanomaterials. Moreover, the development of new methods is needed consistently to analyze the precise mechanical properties and fracture mechanisms.
